# Patient Affected by Beta-Propeller Protein-Associated Neurodegeneration: A Therapeutic Attempt with Iron Chelation Therapy

**DOI:** 10.3389/fneur.2017.00385

**Published:** 2017-08-21

**Authors:** Mattia Fonderico, Michele Laudisi, Nico Golfrè Andreasi, Stefania Bigoni, Costanza Lamperti, Celeste Panteghini, Barbara Garavaglia, Miryam Carecchio, Elia Antonio Emanuele, Gian L. Forni, Enrico Granieri

**Affiliations:** ^1^Department of Biomedical and Specialistic Surgical Sciences, Section of Neurological, Psychiatric and Psychological Sciences, Ferrara University, Ferrara, Italy; ^2^Department of Medical Sciences, Section of Medical Genetics, Ferrara University, Ferrara, Italy; ^3^Unit of Molecular Neurogenetics, Fondazione IRCCS Istituto Neurologico ‘Carlo Besta’, Milan, Italy; ^4^Department of Clinical Neuroscience, Fondazione IRCCS Istituto Neurologico Carlo Besta, Milan, Italy; ^5^Centro della Microcitemia e Anemie Congenite-Haematology, Galliera Hospital, Genoa, Italy

**Keywords:** basal ganglia, NBIA disorders, beta-propeller protein-associated neurodegeneration, iron-chelating agents, iron

## Abstract

Here, we report the case of a 36-year-old patient with a diagnosis of *de novo* mutation of the WDR45 gene, responsible for beta-propeller protein-associated neurodegeneration, a phenotypically distinct, X-linked dominant form of Neurodegeneration with Brain Iron Accumulation. The clinical history is characterized by a relatively stable intellectual disability and a hypo-bradykinetic and hypertonic syndrome with juvenile onset. Genetic investigations and T1 and T2-weighted MR images align with what is described in literature. The patient was also subjected to PET with 18-FDG investigation and DaT-Scan study. In reporting relevant clinical data, we want to emphasize the fact that the patient received a chelation therapy with deferiprone (treatment already used in other forms of NBIA with encouraging results), which, however, had to be interrupted because the parkinsonian symptoms worsened. Conversely, the patient has benefited from non-drug therapies and, in particular, from an adapted motor activity with assisted pedaling (method in the process of validation in treatments of parkinsonian syndromes), which started before the treatment with deferiprone and still continues.

## Introduction

Beta-propeller protein-associated neurodegeneration (BPAN), previously described as static encephalopathy of childhood with neurodegeneration in adulthood, is a genetic disease characterized by neurodegeneration with clear extrapyramidal symptoms, classified among neurodegenerative brain disorders with iron accumulation (NBIA). It is an X-linked disease (X p11.23) caused by *de novo* mutations in the WD repeat domain 45, a gene encoding a beta-propeller scaffold protein [hence the name BPAN (1)] that is considered important for the phenomena of autophagy. Mutations described in literature are mostly *de novo* and most of the affected patients are female, probably because the mutation in homozygosity is not compatible with life, and the affected males would be characterized by somatic mosaicism (2).

Clinical and radiological features of BPAN were already a specific clinical entity before the discovery in 2013 of the responsible genetic mutation (3).

This disorder appears as a delay in the global maturity in childhood and it remains stable up to early adulthood. After a steady clinical situation during adolescence, the patient begins to develop serious parkinsonism together with signs of progressive cognitive impairment with a rapid onset (4, 5).

Other clinical features that have been reported are history of epilepsy, spasticity, Rett-like syndrome features, gaze palsy, sleep disorders, and alterations of the autonomous nervous system (4, 5).

Most of the diagnoses of mutation of the WDR45 gene (including our case) were made after having performed a magnetic resonance imaging (MRI). Pathognomonic of this syndrome is the presence of a hypointensity band on the T2-weighted images at the level of *substantia nigra* and the cerebral peduncle, but less in *globus pallidus*, which distinguishes them from other forms of NBIA (5, 6).

In any case, a definitive diagnosis of this syndrome can only be done through a genetic investigation.

## Patient Presentation

Trying to make data more accessible, they have not been exposed in chronological order but according to clinical criteria.

### Clinical History

The patient (GG) is 36-year-old woman, born by a normal delivery. She lives with her parents and has a brother who is a few years older. She has no family history of neurological diseases. She moved her first steps at the age of 16 months and began to speak her first words when she was 2–3 years old, with evident language deficits. The patient presented a static intellectual disability and had suffered from febrile seizures up to the age of 2. She later presented absence type epileptic seizures with automatic gestures (she repeatedly tapped her thigh), and for this reason, she was treated with phenobarbital, later replaced by vigabatrin and finally by carbamazepine due to allergic reactions. After menarche, she did not have any more seizures and the antiepileptic therapy was suspended.

She obtained a high school diploma with the help of supporting teachers. At present, she is not able to spell, she is able to read single letters, and she can write using a video-writing system. In December 2016, her intelligence quotient (IQ) was 45, a stable value when compared with the last one measured 20 years before when she was 16 years old (IQ 33).

The patient has a hyperprolactinaemia with a globular shape of the adeno-hypophysis and symptomless cysts of the pineal gland.

She has been suffering from movement disorders (bradykinesia and rigidity) for about 10 years but it worsened last year, so she underwent a neurological examination that revealed an increase in the muscle tone, particularly evident in the neck muscles and in the left side of the upper and lower limb; bradykinesia when tapping her both upper limbs; no tremor at rest; negative Romberg; and she had weak, but elicitable, proprioceptive reflexes.

GG featured hypomimic facies with fixed gaze and a dysarthric but intelligible speech. She had an autonomous slow pace when walking although the steps had a normal extent. She had a reduced synkinesias in the upper limbs during gait, a dystonic posture of the upper right limb, and a camptocormic posture. GG presented some stereotypical movements such as pulling down her dress persistently.

In order to slow down the progression of the symptoms, it was decided to promote an iron chelation therapy, similar to what had been done for other forms of NBIA. On July 22, 2016, she started an iron-chelating therapy based on deferiprone in pills, with a dosage of 1,000 mg twice a day. This therapeutic approach was prescribed according to the chelation therapy protocol used by other research groups (7), particularly by Cossu et al. (8).

This therapy was interrupted on November 16, 2016, because there was a significant accentuation of parkinsonian symptoms, a deceleration of the ideomotor activity and, more generally, of the hypo-bradykinetic symptomatology. The worsening started in temporal relation with the beginning of the therapy and continued until its suspension in November 2016. Since then, her parents have noticed a slow and gradual improvement which has taken the patient back to the clinical condition, the state she was before the cure.

**Figure 1:**
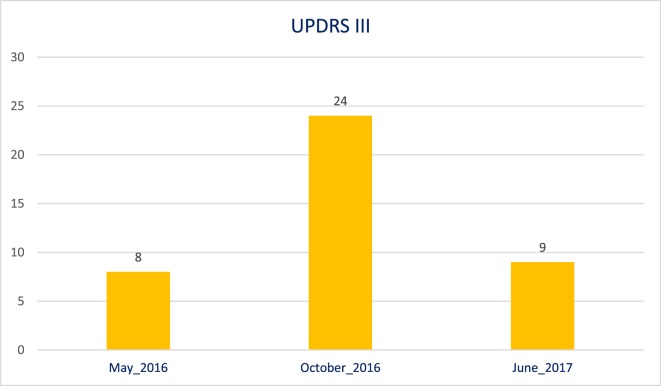
Unified Parkinson’s Disease Rating Scale III evaluation before, during, and after therapy with deferiprone.

This worsening has also been documented through an evaluation with the Unified Parkinson’s Disease Rating Scale (UPDRS III). The UPDRS III had already been performed, during and after the chelation therapy. In the Figure 1, the worsening and also improvement of the patient status can be clearly seen. This phenomenon was also registered in video obtained with the informed consensus of the patient’s parents and is available in three videos: before (Video S1 in Supplementary Material), during (Video S2 in Supplementary Material), and after (Video S3 in Supplementary Material) the deferiprone therapy.

We decided a follow-up program avoiding the dopaminergic treatment on the basis of her mild parkinsonian symptoms.

In the autumn 2015, she started a program of high pedaling cadence forced exercise three times a week from which she benefited in terms of rapidity and accuracy of the movement. Despite this, in the months in which GG took deferiprone, her performances with the bike worsened as well. After the suspension of the therapy, her performances gradually turned back to the previous status and now the patient has reached the levels she had before the treatment. The patient is now taking part in programs of occupational therapy.

The patient has low iron (35 μg/dl) and ferritin (>6 ng/ml) levels in her blood.

### Brain Imaging

**Figure 2:**
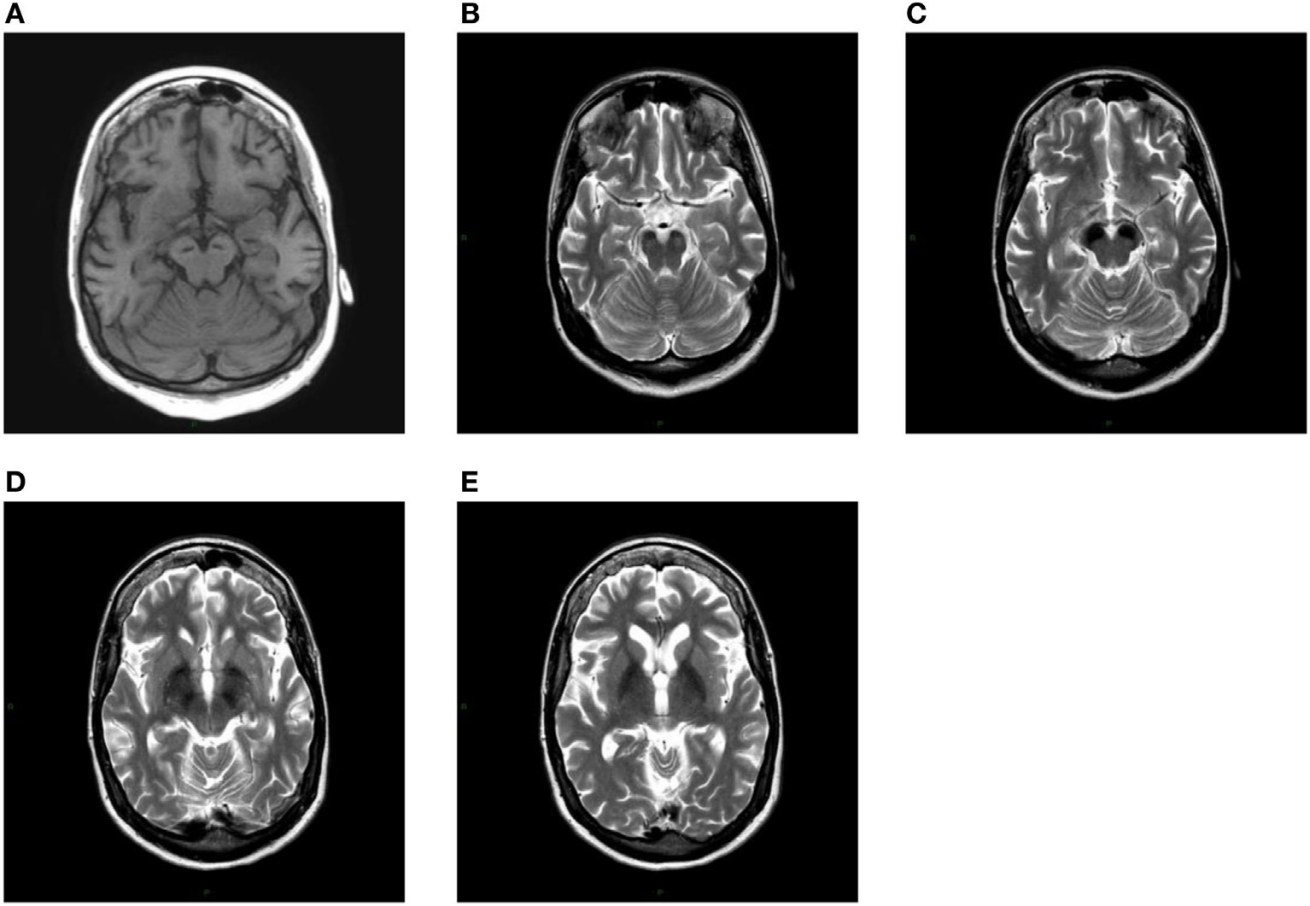
**(A)** T1-weighted axial image shows linear hypointensity at the level of cerebral peduncles in the substantia nigra within a mild area of hyperintensity. At the same level in the images **(B,C)**, both T2-weighted, a striking hypointensity can be seen in both substantiae nigrae. A more cranial axial T2-weighted section shows a marked bilateral hypointensity of the globus pallidus **(D,E)**.

After having found the first extrapyramidal symptoms during a neurological examination, GG was subjected to a first MRI without contrast in 2009, where a prominent signal hypointensity in *substantia nigra* was noticed in the T2-weighted images, indicating the existence of ferromagnetic substances and a cyst in the pineal gland (Figure 2). Two subsequent MRIs were performed in 2015 and in 2016, and they showed an increase in the ferromagnetic deposits in the abovementioned areas.

A brain PET investigation with 18-FDG showed a significant fixing reduction in the radiopharmaceutical in both frontal and left parietal regions.

A brain DaT-Scan was performed in June 2017. It showed a reduction in the uptake of the radio-drug of moderate degree on both putamen and in the *nucleus caudatus*, especially on the left side.

### Genetic Analysis

From the DNA extracted from peripheral blood lymphocytes, 10 exons, encoding the gene WDR 45, were amplified by PCR, and they were sequenced using an automated sequencer (3100 Genetic Analyzer ABI Prism).

This analysis found a c.64DeIT heterozygous mutation in exon 4 of the WDR45 gene, which caused the aminoacid change p.Cys22Alafs*16. We referred Refs (2, 4, 5) and we did not find any previous description of this particular mutation.

## Discussion and Conclusion

The discovery of new mutations that characterize the syndromic picture of NBIAs makes this chapter of neurology increasingly extensive and detailed. In this case report, we tried to report the pathognomonic clinical and neuroimaging features of the disease. T1-weighted image showed a hyperintensity even if it was not as prominent as described by Kruer et al. (6). However, all the other neuroimaging characteristics of this disease were present.

Our patient did not show the cognitive-motor impairments described in other cases. This occurrence could depend on the young age of the patient or be ascribed to a different phenotypic expression of the mutation. The follow-up and collation with literature on this disease may be useful for a future explanation of this case.

In addition, we reported a therapeutic attempt which, as far as we know, had not been reported in BPAN literature before. Therapeutic interventions with deferiprone in NBIA patients have already been conducted with relative success and described in literature (7, 8). However, iron chelation therapy was tested on patients suffering from phantotenate kinase-associated neurodegeneration, the most common form of NBIA. The reason why our patient did not respond positively is not clear, it may depend on unexplained individual characteristics, nevertheless not excluding the intervention of other specific pathophysiological mechanisms in this form of disease. Currently, there is no theoretical base able to explain how deferiprone acts on nigro-striatal transmission. However, animal tests showed that a dose of 100 mg/kg deferiprone is able to reduce DA and 5-HT levels at a striatal plane (9). Future studies will eventually be able to shed light on this.

Regarding patients specifically suffering from mutations of WDR45 (BPAN), they have a discrete response to l-DOPA (5, 7), albeit temporary, taking into account the progressive course of the disease (5). The patient described here was not subjected to l-DOPA therapy as the extrapyramidal symptoms were so mild that a dopaminergic therapy was not justified at such an early stage.

GG benefited from adapted motor activity programs and in particular from the exercises of assisted pedaling three times a week. Assisted pedaling cadence, high–low work rate cycle training of the lower extremities, leads to improvements in walking ability, upper extremities function, balance and QoL in patients suffering from Parkinson’s disease (10). High cadence cycling exercise on an electric motor-driven bike is a simple and effective tool which is ready to be introduced into clinical practice (11).

In future, an increasingly better characterization of these disorders, from a clinical, neuro-radiological, and genetic point of view, will improve diagnoses and personalize therapies in a more efficient way.

## Ethics Statement

No investigations or interventions were performed outside routine clinical care for this patient. As this is a case report, without experimental intervention into routine care, no formal research ethics approval was required. All the diagnostic and therapeutic procedures were obtained with the written, fully informed consent of the patient’s parents. Verbal assent was also given by the patient himself.

## Author Contributions

The authors declare to have given substantial contributions to the conception, acquisition, analysis, and interpretation of the manuscript. All authors have revised the manuscript critically and declared the final approval of the version to be published, and agreed to be accountable for all aspects of the work. MF, MC, and EE were involved in the work-up of the patient and in the planning and conductions of investigations. GF, ML, and NA provided clinical, physical, and psychological care. SB, CL, and BG were involved in genetic analyses. MF drafted the initial manuscript, reviewed and revised the manuscript, and approved the final drafting as submitted. EG coordinated all clinical investigations, critically reviewed the manuscript, implemented some changes to the argument, and approved the final manuscript as submitted.

## Conflict of Interest Statement

The authors declare that the research was conducted in the absence of any commercial or financial relationships that could be construed as a potential conflict of interest. The reviewer, FM, declared a past coauthorship with one of the authors, MC, to the handling editor, who ensured that the process met the standards of a fair and objective review.

## Supplementary Material

Video S1 This video was performed two months before the start of the chelation therapy in the neurological clinic of the University of Ferrara.

Video S2 This video was performed by the mother of the patient in her own home during the chelation therapy. It can be clearly seen the aggravation of the bradykinetic syndrome as well as the more hypomimic face of the patient if compared with the ones in Video S1.

Video S3 This video was registered 4 months after the interruption of the chelation therapy. From the video a reduction of the pendular movements of the upper left arm can be noticed. If compared with Video S2 the bradykinetic syndrome is improved.
